# Obituary Notice of the Late Dr. Enoch Hale

**Published:** 1850-02

**Authors:** 


*Obituary Notice of the late Dr. Enoch Hale.*—Dr. Enoch Hale was too prominent a member of this association to be ever forgotten by those who met him here.

He had the first requisite of a good physician—he was an honest man; there was ever in his mind that fair ingenuousness that seeks for truth alone, and is fearful of all error. He was a careful observer of events, a diligent student of books, and hence an accumulator of many facts. His mind was a rich storehouse of details, out of which he was able to draw wide inferences in regard to the nature of man. Meteorology was his especial favorite, and by his observations of the weather, cold, heat, winds, &c., in connection with health and disease, he endeavored to contribute to the advancement of medical science.

Dr. Hale was early distinguished as a scholar and a writer. Very soon after he began the practice of his profession, he wrote the "History of the Spotted Fever." In 1819 and 1821, he wrote two dissertations "On the communication betwenn the Stomach and the Urinary Organs," and "On the propriety of administerisg Medicine by Injection into the Veins." Each of these obtained the Boylston Prize. In 1839, he delivered the annual address before this Society, on the "Typhoid Fever of New England." Beside these, he was a liberal contributor to the North American Review, and several Medical Journals.

In his intercourse with his patients, in private practice, and "at the hospital, he evinced, in a remarkable degree, that fidelity and tenderness of feeling which gained for him many friends, who regarded him with affection and respect."* In his intercourse with his professional brethren, he showed himself a kind, a just, and a learned member of the profession, and in general society he was a man of distinguished learning, of incorruptible honor, and untiring industry in the walks of usefulness.—*Medical Communications of the Mass. Med. Society.*

*Report of the Trustees of the Massachusetts General Hospital for 1848, p. 5.

